# Exercise facilities and the prevalence of obesity and type 2 diabetes in the city of Madrid

**DOI:** 10.1007/s00125-021-05582-5

**Published:** 2021-10-28

**Authors:** Luis Cereijo, Pedro Gullón, Isabel Del Cura, David Valadés, Usama Bilal, Hannah Badland, Manuel Franco

**Affiliations:** 1 Facultad de Medicina y Ciencias de la Salud, Departamento de Cirugía, Ciencias Médicas y Sociales, Grupo de Investigación en Epidemiología y Salud Pública, Alcalá de Henares, Universidad de Alcalá, Madrid, Spain; 2 Facultad de Medicina y Ciencias de la Salud, Departamento de Ciencias Biomédicas, Grupo de investigación en gestión y entrenamiento deportivo, Alcalá de Henares, Universidad de Alcalá, Madrid, Spain; 3 Centre for Urban Research, RMIT University, Melbourne, VIC Australia; 4 Gerencia de Atención Primaria, Unidad de Investigación de Atención Primaria, Madrid, Spain; 5 Departamento de Especialidades Médicas y Salud Pública, University Rey Juan Carlos, Madrid, Spain; 6 Red de Investigación en Servicios de Salud y Enfermedades Crónicas (REDISSEC) ISCIII, Madrid, Spain; 7 Urban Health Collaborative, Drexel Dornsife School of Public Health, Drexel University, Philadelphia, PA USA; 8 Department of Epidemiology and Biostatistics, Drexel Dornsife School of Public Health, Drexel University, Philadelphia, PA USA; 9 Department of Epidemiology, Johns Hopkins Bloomberg School of Public Health, Baltimore, MD USA

**Keywords:** Electronic medical records, Exercise, Inequities, Obesity, Social determinants, Type 2 diabetes, Urban health

## Abstract

**Aims/hypothesis:**

We aimed to study the association between the availability of exercise facilities and the likelihood of obesity and type 2 diabetes in the adult population of Madrid, Spain.

**Methods:**

We analysed the electronic medical records of all 1,270,512 residents of Madrid aged 40–75 years in 2017. Exercise facility availability was defined as the count of exercise facilities in a 1000 m street network buffer around each residential building entrance. Poisson regression with standard errors clustered at census tract level was used to assess prevalence ratios of exercise facility availability tertiles and obesity and type 2 diabetes. We also examined stratified results by tertiles of area-level socioeconomic status (SES) and sex.

**Results:**

People living in areas with lower availability of exercise facilities had a higher prevalence of obesity (prevalence ratio [PR] 1.22 [95% CI 1.20, 1.25]) and diabetes (PR 1.38 [95% CI 1.34, 1.43]). We observed effect modification by area-level SES (*p*<0.001), with stronger associations for residents living in low-SES areas and no association for residents living in high-SES areas. Associations with type 2 diabetes were stronger among women compared with men, while associations with obesity were similar by sex.

**Conclusions/interpretation:**

People living in areas with low availability of exercise facilities had a higher prevalence of obesity and type 2 diabetes, and this association was strongest in low-SES areas and for women. Understanding the potential role of exercise facilities in driving inequities in obesity and type 2 diabetes prevalence may inform interventions to reduce health inequities.

**Graphical abstract:**

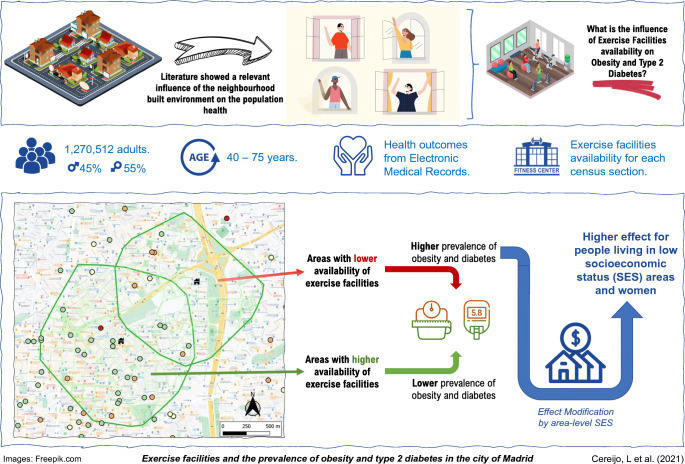

**Supplementary Information:**

The online version contains peer-reviewed but unedited supplementary material available at 10.1007/s00125-021-05582-5.

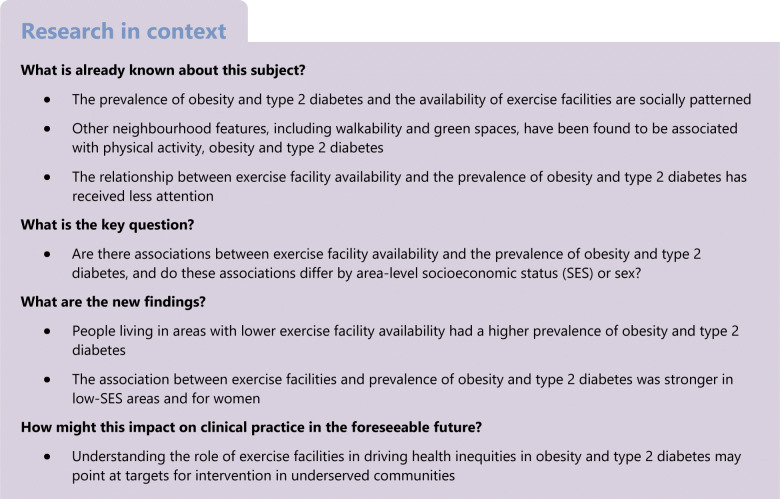



## Introduction

Increased physical activity is linked to reductions in risks of type 2 diabetes and obesity [1–3]. Neighbourhood features (e.g. parks, green spaces, physical activity facilities) are important determinants of physical activity [4], especially in more disadvantaged neighbourhoods [5, 6]. Population-level approaches targeting neighbourhood environment features may be promising strategies to address type 2 diabetes and obesity [7, 8], especially in light of existing type 2 diabetes [9] and obesity [10] social gradients. For example, social determinants of health are key drivers of type 2 diabetes and related outcomes [11].

Increased physical activity opportunities at the neighbourhood level are associated with lower obesity [12] and type 2 diabetes risk [13]; for example, higher availability of green and open spaces is associated with lower type 2 diabetes prevalence [14, 15] and incidence [16, 17]. However, few studies have investigated relationships between the availability of exercise facilities and obesity and type 2 diabetes [12, 18]. Exercise facility programmes tend to be structured and occur at moderate to vigorous intensity [19], eliciting health benefits [20]. Therefore, higher availability of exercise facilities potentially increases opportunities for structured exercise, which is associated with a lower prevalence of obesity [12] and greater reductions in HbA_1c_, compared with delivering physical activity advice alone [21].

We previously demonstrated a social gradient for exercise facility availability in Madrid [22] and for type 2 diabetes prevalence, incidence and control [9]. Previous research has shown that amenities conducive to physical activity, including parks and green spaces, can reduce health inequities [23]. Moreover, there has been limited research exploring exercise facility differences by sex, and the little available evidence shows that women are less likely to use exercise facilities than men [24]. Thus, examining relationships between exercise facilities and type 2 diabetes and obesity by area-level socioeconomic status (SES) and sex can help identify potential interventions to address these inequities by focusing on populations most in need.

The study aims were as follows: (1) to examine the association between availability of exercise facilities and the likelihood of obesity and type 2 diabetes in the adult (40–75 years old) population of Madrid; and (2) to examine interactions with area-level SES and sex.

## Methods

### Study design

A population-based retrospective cohort study using data from primary care electronic medical records (EMRs) in Madrid, Spain was conducted. This study was developed based on the REporting of studies Conducted using Observational Routinely-collected Data (RECORD) statement [25]. The study followed a multilevel design using variables at the individual (age, sex, obesity and diabetes) and neighbourhood level (population density, SES and exercise facility availability).

### Setting

This study is part of the Heart Healthy Hoods (HHH) project, which broadly aims to study associations of the social and physical urban environment with cardiovascular health and inequity in Madrid, Spain [26]. This study was conducted across the municipality of Madrid. In 2017 Madrid had a population of 3.2 million residents and it is divided into 21 districts that are composed of 128 neighbourhoods. Within each neighbourhood there are small geographical administrative units of ~1500 people each, called census tracts (*secciones censales*) (*N*=2415) [27]. Further information about the demographic composition of the administrative units in Spain is shown in electronic supplementary material (ESM) Table 1.

### Study population

The HHH cohort is based on real-world data from primary care, including information about 1,305,050 residents. The individuals in the HHH cohort represented 91% of the total population of the age group included in this study (40–75 years) living in Madrid [27]. The study population was selected according to the HHH project criteria [28] as individuals: (1) registered at one of the 128 primary healthcare centres in the municipality of Madrid; (2) who live in the municipality of Madrid; (3) aged 40–75 years; (4) registered in the EMRs of the Primary Health-care Service of Madrid (AP-MADRID) in 2017, with no missing data for obesity and/or diabetes.

### Health outcomes

Diagnoses (recorded by primary care physicians during their usual clinical care) were extracted from EMRs for all individuals. These diagnoses were coded according to the International Classification of Primary Care (ICPC-2; www.who.int/standards/classifications/other-classifications/international-classification-of-primary-care). Type 2 diabetes was defined using the T90 diagnosis code (‘diabetes non-insulin dependent’). Type 2 diabetes diagnoses in the Primary Health-care Service of Madrid dataset have been previously validated with a κ of 0.99, with a sensitivity of 99.5% and a specificity of 99.5% [29]. Obesity was defined as BMI ≥30 kg/m^2^ and was objectively measured.

### Exercise facilities

Exercise facilities were defined as venues that offered exercise programmes, whether free, monthly subscription or pay per session (e.g. fitness clubs, sports centres, dance clubs, Pilates studios), and regardless of whether they were publicly or privately owned. Exercise facility information was collected by ‘MAS Servicios Integrales’, a fitness consultancy firm, between April and October of 2015. All exercise facilities meeting these criteria across Madrid were identified using Google Maps. Information about the programmes and services was sourced through telephone and face-to-face interviews with facility managers. More information about data collection can be found elsewhere [22]. The final exercise facility dataset comprised 595 facilities with information collected for five characteristics: (1) facility name; (2) facility physical address; (3) monthly price; (4) types of programmes and services offered; (5) ownership (public vs private) (see ESM Table 2).

### Availability measures

**Fig. 1:**
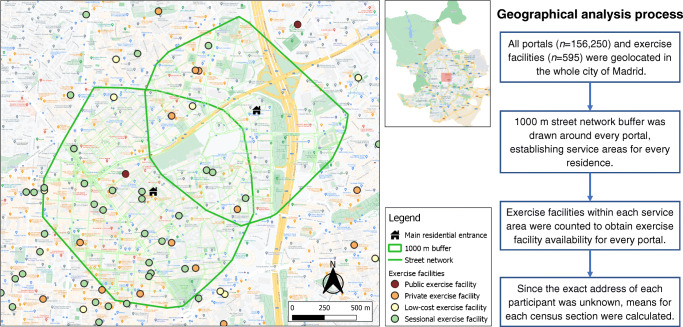
Diagram of the geographical analysis to calculate the availability of exercise facilities

We captured residential building entrances (hereafter called portals) in Madrid (Fig. 1). This was done by identifying all external access points to residences located in residential land use using the GEOPORTAL of the Madrid City Council [30]. Spatial measures were calculated using QGIS 3.10.5 software. Based on the definition by Penchansky and Thomas [31], exercise facility availability was calculated as the count of facilities within a 1000 m street network buffer from each portal. All portals in a census tract were aggregated and a mean count of exercise facilities for each census tract was calculated. The 1000 m buffer has been used in exercise facility research [12, 22, 32, 33] and is regarded as an appropriate walking distance for undertaking daily activities [34]. Also, a 1000 m street network distance from home to an exercise facility showed the highest correlation with moderate to vigorous physical activity [32]. Census tracts were stratified into tertiles of exercise facility availability. Sensitivity analyses using deciles of exercise facility availability were also conducted. Boundaries of the geographic information data were from 1 January 2017.

### Area-level SES

Area-level SES was obtained from a composite area-level SES index created using seven SES indicators: (1) low education; (2) high education; (3) part-time employment; (4) temporary employment; (5) manual occupational class; (6) average housing prices (per m^2^); and (7) unemployment rate. The SES index was calculated for each census tract across Madrid and was collapsed into tertiles (low, medium, high). The index has been used in previous studies [9, 22], and further details regarding index construction are described in ESM Table 3.

### Statistical analysis

The analysis was undertaken in three steps. First, key demographic and clinical characteristics of the population were described. Second, Poisson regression models with robust standard errors clustered at the census tract level were applied to estimate the prevalence ratio (PR) for the association between the exposure (exercise facilities) and each outcome (type 2 diabetes, obesity). We initially created an unadjusted model (Model 0), then adjusted the model by age and sex (Model 1), together with population density (Model 2), and finally adjusted by area-level SES (Model 3). Third, to identify any potential effect modification from area-level SES with the availability of exercise facilities, we introduced an interaction term between area-level SES and availability of exercise facilities, using a Wald test to get a *p* value for each interaction. Results were presented for the whole sample and stratified by sex, using the highest tertile of exercise facilities available as the reference. All analyses were conducted using Stata/SE 14.1 for Mac (StataCorp, College Station, TX, USA).

### Ethics

This study was carried out under the umbrella of the HHH study and in accordance with the Declaration of Helsinki guidelines. The study received Institutional Review Board (IRB) approval from the Ethics Research Committee of the Madrid Health Care System on 12 May 2015.

## Results

After excluding those with missing data on residential location (*n*=34,538), our final sample included 1,270,512 individuals for the type 2 diabetes analysis and 213,719 adults for the obesity analysis. The distributions by area-level SES and availability of exercise facilities of participants between those with and without missing values of obesity were similar. Table 1 shows the final sample characteristics. A social gradient was evident for type 2 diabetes, whereby type 2 diabetes was more prevalent in low-SES areas (9.1%), compared with medium- (7.1%) and high-SES (5.0%) areas, and in men (8.6% compared with 5.8% in women). Obesity presented a similar distribution to type 2 diabetes. People living in lower-SES areas had a higher prevalence of obesity (43.7%) when compared with medium- (37.7%) and higher-SES areas (30.6%). Men had higher prevalence of obesity (39.4%) than women (37.5%). Availability of exercise facilities also showed a social gradient (low [median facilities = 5; IQR 3–8], medium [median = 7; IQR 4–12] and high SES [median = 12; IQR 4–18]).

**Table 1:** Characteristics of the study sample

Characteristic	Overall		High availability of exercise facilities^a^		Medium availability of exercise facilities^a^		Low availability of exercise facilities^a^	
	Men	Women	Men	Women	Men	Women	Men	Women
*n*	574,440	696,072	186,071	237,381	191,709	230,864	196,660	227,827
Age, years	51.0 (45.0–60.0)	53.0 (46.0–63.0)	52.0 (45.0–61.0)	54.0 (47.0–64.0)	52.0 (45.0–60.0)	53.0 (46.0–63.0)	51.0 (45.0–59.0)	52.0 (45.0–62.0)
Area-level SES, index value	−0.200 (−0.783–0.649)	−0.160 (−0.754–0.678)	0.554 (−0.201–0.805)	0.593 (−0.176–0.825)	−0.582 (−1.015–0.061)	−0.561 (−0.993–0.063)	−0.459 (−1.032–0.556)	−0.454 (−1.019–0.563)
Population density, habitants/km^2^	30,000 (17,400–43,000)	30,200 (17,600–43,300)	39,600 (26,700–52,900)	39,500 (26,700–52,800)	30,800 (20,000–41,400)	30,700 (20,000–41,300)	20,400 (9420–32,900)	20,400 (9420–32,900)
Exercise facilities, *n*	6.57 (3.13–11.59)	6.90 (3.30–12.08)	14.44 (11.81–19.67)	14.51 (11.90–19.87)	6.72 (5.50–8.08)	6.77 (5.53–8.10)	2.26 (1.39–3.21)	2.29 (1.39–3.21)
Type 2 diabetes	49,458 (8.6)	40,247(5.8)	14,674(7.9)	11,781 (5.0)	17,604 (9.2)	14,521 (6.3)	17,180 (8.7)	13,945 (6.1)
Obesity^b^	34,721 (39.4)	47,056 (37.5)	9265 (36.3)	12,543 (33.2)	12,507 (40.2)	17,214 (38.6)	12,949 (41.0)	17,299 (40.1)

Data displayed are median (IQR) or *n* (%)

^a^Exercise facility availability was defined as count of exercise facilities in a 1000 m street network buffer around each portal, and divided into high, medium and low tertiles

^b^A subgroup of *n*=213,719 individuals (*n*=88,224 men and *n*=125,495 women) was included in the obesity analysis

We found a significant relationship of exercise facility availability with obesity and type 2 diabetes prevalence: people living in areas at the lowest tertile of exercise facility availability had a significantly higher prevalence of obesity (PR_Tertile 3 vs 1_ = 1.22 [95% CI 1.20, 1.25]) and type 2 diabetes (PR_Tertile 3 vs 1_ = 1.38 [95% CI 1.34, 1.43]). However, these associations were attenuated, but remained significant, after adjustment by area-level SES (Model 3; obesity, PR_Tertile 3 vs 1_ = 1.03 [95% CI 1.01, 1.05]; type 2 diabetes, PR_Tertile 3 vs 1_ = 1.03 [95% CI 1.00, 1.06]; see Table 2). Models 2 and 3 show the independent effects of exercise facility availability on the prevalence of obesity and type 2 diabetes. The independent effect for the third tertile (higher availability of exercise facilities) is 14% (PR 1.03 vs the total effect of PR 1.22) for obesity and 8% (PR 1.03 vs the total effect of PR 1.38) for type 2 diabetes.

**Table 2:** Association of exercise facility availability with prevalence of obesity and type 2 diabetes in Madrid

Exercise facility availability	Model 0: crude	Model 1: adjusted by age and sex	Model 2: adjusted by age, sex and population density	Model 3: adjusted by age, sex, population density and SES
Obesity				
High density	1 (Ref.)	1 (Ref.)	1 (Ref.)	1 (Ref.)
Medium density	1.14 (1.12, 1.16)**	1.14 (1.11, 1.16)**	1.17 (1.14, 1.19)**	1.00 (0.98, 1.02)
Low density	1.17 (1.15, 1.20)**	1.17 (1.15, 1.20)**	1.22 (1.20, 1.25)**	1.03 (1.01, 1.05)**
Type 2 diabetes				
High density	1 (Ref.)	1 (Ref.)	1 (Ref.)	1 (Ref.)
Medium density	1.22 (1.18, 1.25)**	1.24 (1.20, 1.27)**	1.29 (1.25, 1.33)**	0.98 (0.95, 1.00)*
Low density	1.17 (1.13, 1.22)**	1.27 (1.23, 1.32)**	1.38 (1.34, 1.43)**	1.03 (1.00, 1.06)*

Data are presented as PR (95% CI)

*p< 0.05, **p< 0.01

Ref., tertile of reference

### Effect modification of area-level SES and sex

**Fig. 2:**
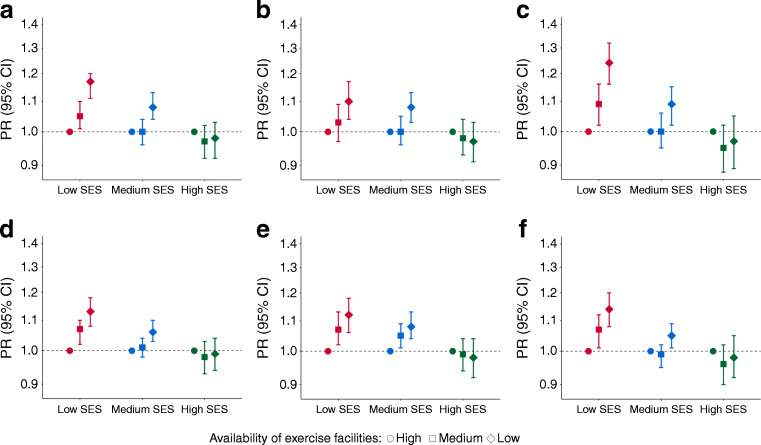
Association of exercise facility availability with (**a**–**c**) type 2 diabetes and (**d**–**f**) obesity, adjusted by age and population density. Interactions by area-level SES are presented. Overall data are shown (**a**, **d**), as well as data stratified by sex: men (**b**, **e**); women (**c**, **f**). Dashed lines at PR 1.0 represent the reference group (highest availability of exercise facilities). There was a statistically significant effect modification of area-level SES on the relationship between exercise facility availability and obesity and type 2 diabetes (*p* value for interaction <0.001)

Figure 2 shows a statistically significant effect modification of area-level SES on the relationship between exercise facility availability and obesity and type 2 diabetes (*p* value for interaction <0.001). For those living in the lowest area-level SES, a lower availability of exercise facilities was associated with a higher prevalence of obesity (PR_Tertile 3 vs 1_ = 1.13 [95% CI 1.08, 1.18]) and type 2 diabetes (PR_Tertile 3 vs 1_ = 1.17 [95% CI 1.11, 1.20]). We found no association between exercise facility availability and the prevalence of obesity or type 2 diabetes for people living in high-SES areas. When stratified by sex, we found a stronger association between exercise facility availability and type 2 diabetes for women (PR_Tertile 3 vs 1_ = 1.24 [95% CI 1.16, 1.32]) compared with men (PR_Tertile 3 vs 1_ = 1.10 [95% CI 1.04, 1.17]).

### Sensitivity analysis

Analysis by deciles of exercise facility availability showed a linear and gradual association between facility availability and both health outcomes, with the exception of the highest decile (see ESM Table 4).

## Discussion

In this study of ~1.3 million adults in Madrid (Spain), we found that residents living in areas with lower availability of exercise facilities had higher levels of obesity and type 2 diabetes. These associations were strongest in lower-SES areas. To our knowledge, this is the first study exploring exercise facility availability and obesity and type 2 diabetes associations through area-level SES effect modification.

These results are consistent with previous studies that described higher prevalence of obesity [12] and type 2 diabetes [18] in residents living in areas with lower availability of exercise facilities. Importantly, we found that the association between exercise facility availability and the prevalence of obesity and type 2 diabetes was largely attenuated after adjusting for area-level SES. This has two important implications. First, it indicates that part of the social gradient in obesity and type 2 diabetes prevalence may be explained by the differential distribution of exercise facilities. A potential pathway of these associations may be through increased physical activity; previous studies in Madrid with older adults (50–74 years) showed that availability of exercise facilities was a mediator between neighbourhood economic context and physical inactivity [35]. Second, these patterns indicate that areas with the highest prevalence of obesity and type 2 diabetes are areas characterised by low SES and with a low availability of exercise facilities. These findings may have potential policy implications as they indicate that exercise facilities may be able to partially mitigate SES inequities.

In the stratified models, prevalence of obesity and type 2 diabetes was greater among people who lived in lower-SES areas and with a lower level of exercise facility availability. Taken together, a lack of exercise facilities may contribute to the social gradient among more deprived populations. Investigating the interaction of characteristics of the built environment and SES is crucial to understanding the extent of health inequities and designing potential interventions to prevent these inequities [36].

Sex inequities were also identified. The magnitude of obesity and type 2 diabetes PRs was higher among women from low-SES areas and with low availability of exercise facilities, when compared with the equivalent male population. When type 2 diabetes was considered, the PR for women living in low-SES areas with low availability of exercise facilities was double that for men living in areas with the same characteristics. Similar sex inequities have been reported in research examining adiposity and availability of exercise facilities [12], and green space availability and diabetes [37].

A strength of the study is including the entire adult population of a major European city (Madrid) where almost 1,400,000 adults live [28]. This large sample size minimised selection bias compared with surveys or regular cohort studies [38], and allowed us to capture geographic and demographic variation across a city. The diagnosis of type 2 diabetes in our EMRs was previously shown to have high validity [29] and obesity was classified objectively.

The current study also presents several limitations. First, cross-sectional studies of neighbourhood environment are prone to reverse causation, as individuals with lower BMI may choose to live in areas with more exercise facilities. Although self-selection bias is a concern in neighbourhood and health studies, its effect is not clear and should be confirmed in future studies [39, 40]. Second, the study did not consider the physical activity levels of participants, so we cannot confirm whether presence of exercise facilities is associated with exercise facility use. However, studies have found that greater availability of exercise facilities in neighbourhoods was associated with higher levels of overall physical activity [35, 41, 42]. Third, since our focus was on indoor exercise facilities, it is likely we missed other physical activity destinations, such as outdoor sports courts, parks and pavements/footpaths. Fourth, the measurement of our outcomes relied on EMR data, which may be subject to bias. However, a validation study conducted using these same datasets found type 2 diabetes diagnoses accurate (κ = 0.99). We have no information on the validity of the diagnosis of obesity. Finally, there were temporal differences across the datasets (2015, 2017 for EMRs; 2017 for area-level SES). Although the area-level SES has not changed significantly over the last few years, it is possible that variations in the exercise facilities have not been captured.

### Research agenda

Study findings opened two lines of inquiry for improving our understanding of the associations between exercise facilities and health outcomes. Future studies should seek to confirm the results presented in this research using individual-level behavioural data captured through longitudinal studies to better understand how the presence of exercise facilities is associated with facility use and physical activity engagement, and related inequities. Integrating qualitative methods to evaluate the characteristics of exercise facilities would be helpful to gain a better understanding of barriers and enablers for using exercise facilities, and whether these differ by sex and SES.

### Policy recommendations

Our study showed the highest prevalence of obesity and type 2 diabetes in low-SES areas with the lowest availability of exercise facilities. This finding suggests that obesity and type 2 diabetes prevention efforts should focus in these areas to reduce health inequities. Preventive efforts should also include mechanisms to reduce sex inequities in access to exercise facilities, as we found stronger associations with type 2 diabetes for women.

### Conclusions

Our findings from ~1.3 million adult Madrid residents demonstrated that neighbourhoods with lower availability of exercise facilities had a higher prevalence of obesity and type 2 diabetes, and this was most evident for women and for people living in low-SES neighbourhoods. These findings provide knowledge that may help inform interventions to reduce health inequities.

## Supplementary Information

ESM 1 (PDF 202 kb)

## Data Availability

The datasets generated during and/or analysed during the current study are not publicly available due to ethical restrictions but are available from the researchers of the HHH project Manuel Franco (manuel.franco@uah.es) and Isabel del Cura (isabel.cura@salud.madrid.org) on reasonable request.

## Abbreviations

EMR: Electronic medical record
HHH: Heart Healthy Hoods
PR: Prevalence ratio
SES: Socioeconomic status
