# Supplementary material for: Exercise facilities and the prevalence of obesity and type 2 diabetes in the city of Madrid
Source: Diabetologia. 2021 Oct 28;65(1):150–8. doi: 10.1007/s00125-021-05582-5 (PMC8660723; doi:10.1007/s00125-021-05582-5)
Supplement: Supplementary file 1 — (PDF 202 kb) [file 125_2021_5582_MOESM1_ESM.pdf]

Exercise facilities and prevalence of obesity and type 2 diabetes mellitus: A population study of 1,270,512 adults from an equity perspective in the city of Madrid.

Luis Cereijo, Pedro Gullón, Isabel del Cura, David Valadés,
Usama Bilal, Hannah Badland, Manuel Franco.

SUPLEMENTARY MATERIALS

ESM Table 1. Characteristics by January 1st, 2017 of administrative units in the City of Madrid and whole Spain.

ESM Table 2. Description of the classification of the exercise facilities based on its characteristics.

ESM Table 3. Area Level Socioeconomic status indicators.

ESM Table 4. Sensitivity analysis by deciles of exercise facilities availability.

ESM Table 1. Characteristics by January 1st, 2017 of administrative units in the City of Madrid and whole Spain

	Unit	Description	N	Area*	Population*
SPAIN	**Autonomous Communities**	Main Regional division of Spain	17	11073.55 (4995.87-93827.15)	2031.48 (315.38-8379,82)
	**Provinces**	Main Regional Subdivision	52	9722.31 (1906.09-21792.47)	611764.5 (84.96-6507.18]
	**Municipalities**	Main Local division of Spain	8124	34.9 (0.03-1753.85)	0.53 (0-3182.98)
CITY OF MADRID	**Census Districts**	Main Local subdivision	21	14.05 (4.68-237.84)	143.42 (46.88-244)
	**Neighborhoods**	Sub-divisions of districts	131	1.36 (0.25-187.6)	22.40 (1.11-80.3)
	**Census Sections**	Basic census area	2443	0.04 [0.1-94.7]	1.2 (0.11-2.44)

Key: *Area is in km^2 and shown as the Median (Min-Max); Population is in 1000s of residents, excluding the two Autonomous Cities (Ceuta and Melilla), and shown as the Median (Min-Max).

ESM Table 2. Description of the classification of the exercise facilities based on its characteristics.

Exercise facility type	Definition	*N (%)*
**All the facilities**		595
**Publicly owned**	Monthly payment option. Public ownership	59 (10%)
**Privately owned**	Monthly payment $\geq$ 30€/month. Private ownership	222 (37%)
**Low cost**	Monthly payment < 30€/month. Private ownership	63 (11%)
**Sessional**	Facilities with Pay-per-session (e.g. Pilates Studios, Dance Schools, electrostimulation centres…). Private ownership.	251 (42%)

ESM Table 3. Area Level Socioeconomic status indicators.

Construct	Domain	Indicator	Operationalization	Source	Level
SES	Education	Low Education	Residents with mandatory studies or below / all residents aged 25 years or above	Padron	Census Section
		High Education	Residents with university education or above / all residents aged 25 years or above	Padron	Census Section
	Occupation	Part time Jobs	Workers in part-time jobs / all workers	Social Security	Neighbourhood
		Temporary Jobs	Workers in temporal jobs / all workers		
		Manual Occupation Class	Workers in manual or unskilled occupations / all workers		
	Wealth	Housing Prices	Average sale price of housing per m^2	Idealista Report	Census Section
	Living Conditions	Unemployment Rate	Residents registered as unemployed / all residents aged 16–64 years	Employment Service	Neighbourhood

Key: SES = Socio-Economic Status

ESM Table 4. Sensitivity analysis: Association between exercise facility availability (by deciles) and prevalence of obesity and type 2 diabetes in Madrid, adjusted by gender, age and population density.

**Exercise facilities availability**	**Decil 1** High	**Decil 2**	**Decil 3**	**Decil 4**	**Decil 5**	**Decil 6**	**Decil 7**	**Decil 8**	**Decil 9**	**Decil 10** Low
**Prevalence Ratio of Obesity**	1 (Ref.)	1.08 (1.05 - 1.11)	1.09 (1.06 - 1.12)	1.19 (1.16 - 1.22)	1.22 (1.19 - 1.26)	1.26 (1.23 - 1.30)	1.30 (1.26 - 1.33)	1.30 (1.26 - 1.33)	1.36 (1.33 - 1.40)	1.26 (1.22 - 1.29)
**Prevalence Ratio of Type 2 Diabetes**	1 (Ref.)	1.13 (1.09 - 1.16)	1.22 (1.18 - 1.26)	1.43 (1.39 - 1.47)	1.41 (1.37 - 1.45)	1.54 (1.49 - 1.58)	1.55 (1.51 - 1.60)	1.57 (1.52 - 1.61)	1.69 (1.64 - 1.74)	1.49 (1.44 - 1.54)

Results are Prevalence Ratio (Confidence Interval at 95%). All coefficients are statistically significant (p<0.000). Ref= Decile of reference.
